# Investigating the Immune Basis of Green Tea Extract Induced Liver Injury in Healthy Donors Expressing HLA-B*35:01

**DOI:** 10.1021/acs.chemrestox.3c00253

**Published:** 2023-12-06

**Authors:** James Line, Serat-E Ali, Sophie Grice, Tai Rao, Dean J. Naisbitt

**Affiliations:** † Department of Pharmacology and Therapeutics, University of Liverpool, Sherrington Building, Ashton Street, Liverpool L69 3GE, United Kingdom; ‡ Department of Clinical Pharmacology, Xiangya Hospital, Central South University, Changsha 410008, China; § Hunan Key Laboratory of Pharmacogenetics, Institute of Clinical Pharmacology, Central South University, Changsha 410008, China

## Abstract

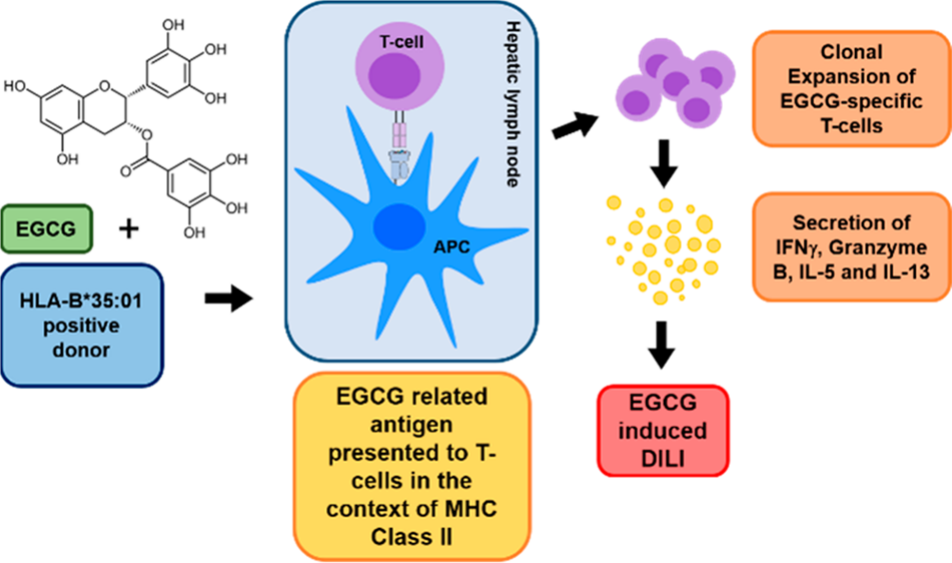

Epigallocatechin-3-*O*-gallate (EGCG) is the major component of green tea extract, commonly found in dietary supplements, and has been associated with immune-mediated liver injury. The purpose of this study was to investigate the immunogenicity of EGCG in healthy donors expressing HLA-B*35:01, and characterize EGCG responsive T-cell clones. We have shown that EGCG can prime peripheral blood mononuclear cells and T-cells from donors with and without the HLA-B*35:01 allele. T-cell clones were CD4^+ve^ and capable of secreting Th1, Th2, and cytolytic molecules. These data demonstrate that EGCG can activate T-cells *in vitro*, suggesting a significant role in the pathogenesis of green tea extract induced liver injury.

Herbal and dietary supplements (HDSs) have become a frequent component of daily life for millions of people across the globe, with over 50% of the US population taking at least one HDS per day.^1^ HDSs account for 20% of drug-induced liver injury (DILI) in the US, with the increased usage of HDSs over the last few decades correlating with an increase in the proportion of DILI cases that are attributable to HDSs.^2^ Green tea extract (GTE) is derived from the *Camellia sinensis* plant and is a common constituent of multi-ingredient HDSs. Since 2006, GTE has been linked to over 50 cases of idiosyncratic liver injury.^3−6^ GTE-DILI is characterized by hepatocellular liver damage (with increases in serum transaminases) and is fatal in around 9% of cases.^4^ Features of the reactions including a delayed onset, resolution of clinical symptoms following cessation of GTE intake, and rapid reoccurrence of symptoms upon rechallenge suggests they may be immune mediated.^4^

There are multiple major catechins present in GTE, with (−) epigallocatechin-3-*O*-gallate (EGCG) being the most abundant, making up between 5 and 12% of the bioavailable catechins.^7,8^ EGCG is among several herbal derived flavonoid compounds that have a strong association with the expression of HLA-B*35:01 and hepatocellular or cholestatic liver injury. 72% of confirmed GTE-DILI patients express the risk allele, which is otherwise present in the population at between 5 and 15%.^4^

In vivo studies have been conducted in programmed cell death protein 1 (PD-1)^(−/−)^ murine models, which demonstrated that at doses considered safe, GTE can drive increased ALT levels and increase the population of cytotoxic T-cells present in the liver.^9^

The aim of this study was to investigate the immunogenicity of EGCG using *in vitro* techniques, utilizing peripheral blood mononuclear cells (PBMCs) and T-cells from healthy donors, in addition to interrogating the allele restriction of any responses observed.

PBMCs from HLA-B*35:01 positive and negative healthy donors were cocultured with EGCG (25 μM) for 3 weeks and restimulated every 7 days with irradiated autologous PBMC, EGCG (25 μM), and IL-2 before being washed and rechallenged with various concentrations of EGCG (1–50 μM). Priming of PBMC to EGCG was identified in 2 of 4 allele positive donors and 1 of 4 allele negative donors, with statistically significant (*p* < 0.0001) proliferation in response to EGCG treatment (Figure 1A). Additionally, secretion of T_h_1, T_h_2, and cytolytic molecules (IFN-γ, IL-5, IL-13, and Granzyme B) was observed (Figure 1B).

**Figure 1:**
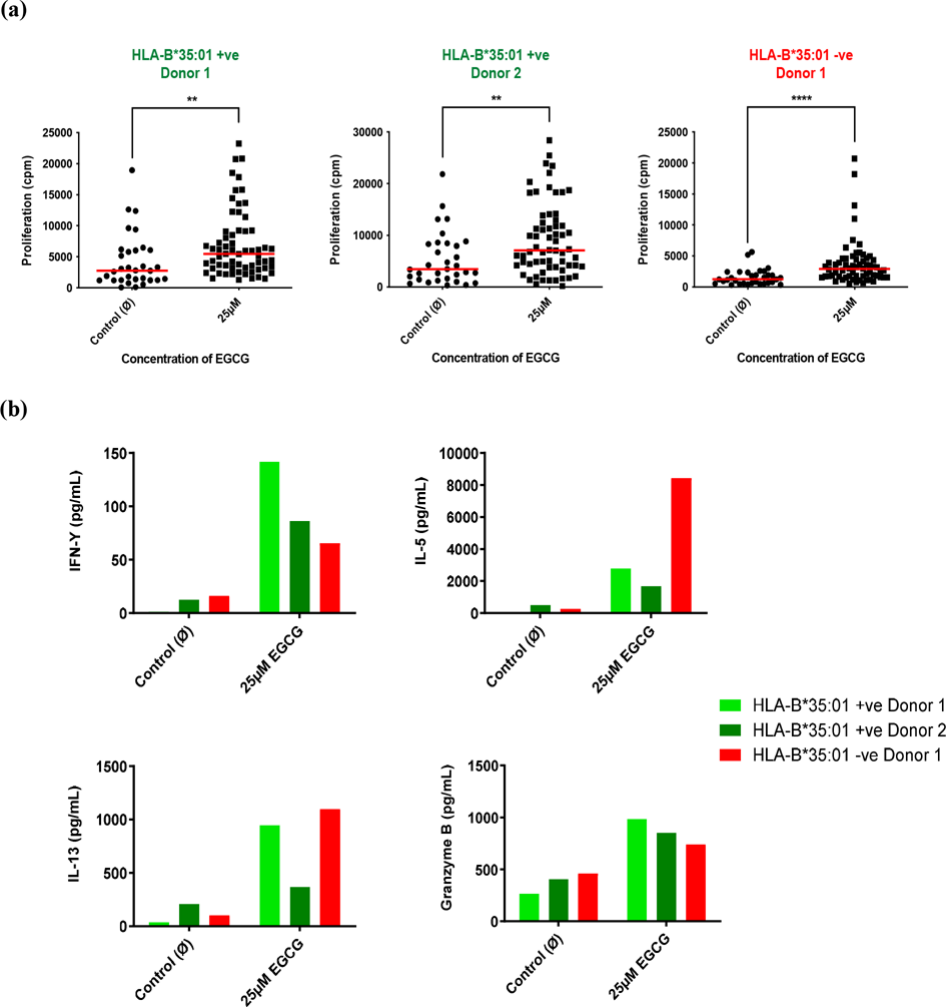
(a) Priming of PBMC isolated from HLA-B*35:01 positive (green) and HLA-B*35:01 negative (red) healthy donors. EGCG (25 μM) and PBMCs were cocultured for 3 weeks and restimulated every 7 days with irradiated autologous PBMCs, EGCG (25 μM), and IL-2. Cells were washed on day 21 and rechallenged with either EGCG or cell culture medium for 48 h. (a) A proliferation reading was taken, through addition of ^3^H thymidine for the final 16 h. Statistical significance was determined using a Mann–Whitney test (**p < 0.01, ****p < 0.0001). (b) Cytokine secretion was measured from assay supernatant pooled from each condition using cytometric bead-based immunoassays (LEGENDplex, BioLegend Custom Human 11-plex panel) carried out per manufacturer’s instructions using a BD FACS Canto II.

Furthermore, T-cells isolated from random (non-HLA typed) healthy donors were cocultured with autologous monocyte derived dendritic cells (moDCs) and EGCG (25 μM) in the presence or absence of anti-PD-1 or programmed cell death ligand 1 (PD-L1) blocking antibodies (5 μg/mL). After 12 days, cells were washed and rechallenged with EGCG (25 μM) and a proliferation reading was taken. Priming of T-cells was enhanced in the presence of PD-1 and PD-L1 blockade, with stimulation index (SI) figures of over 10 in conditions subject to immune checkpoint blockade, compared to a maximum SI of 4 in unblocked conditions (Figure 2A,B). These data suggest that EGCG is immunogenic in nature and is able to prime PBMCs and T-cells from allele positive and negative healthy donors.

**Figure 2:**
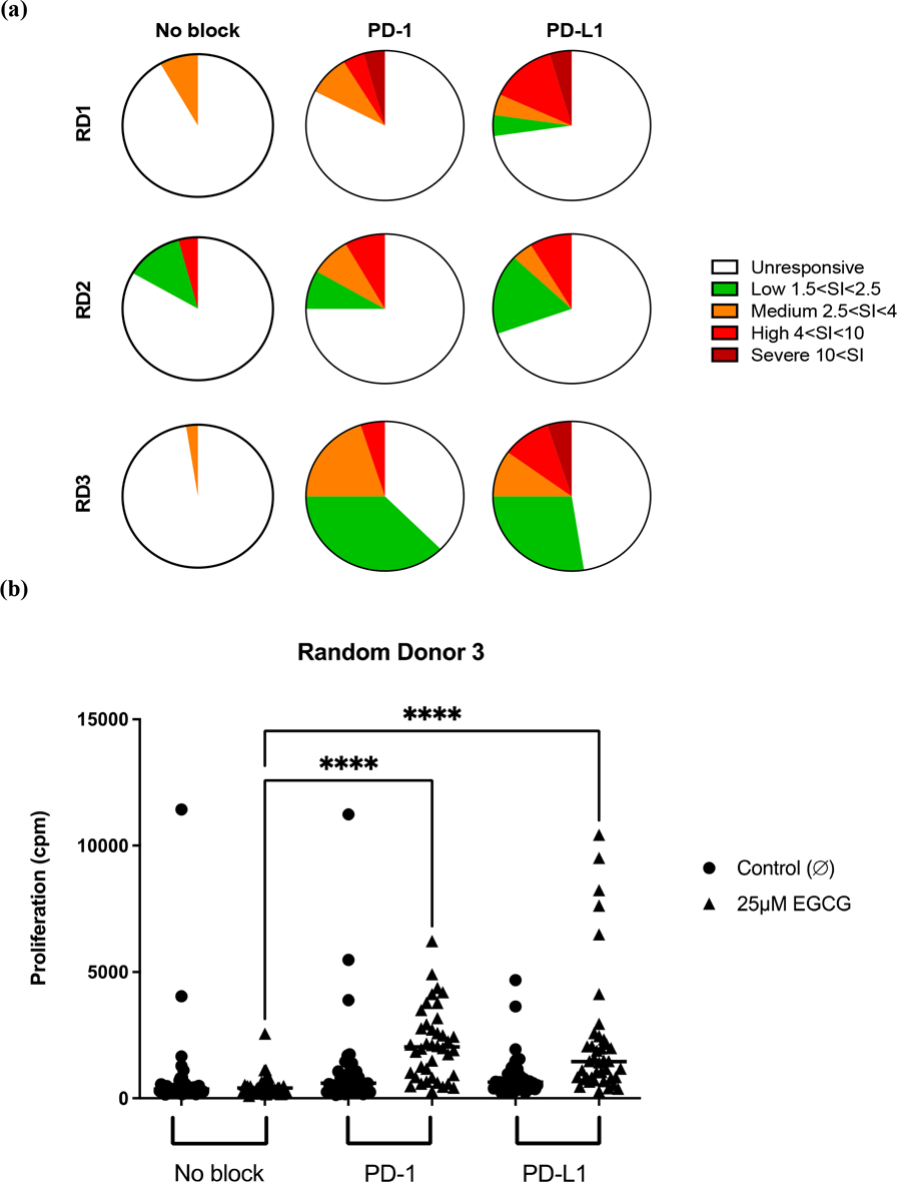
Priming of T-cells isolated from random healthy donors. (a) T-cells and monocyte derived dendritic cells (moDCs) were cocultured with EGCG (25 μM) in the presence or absence of PD-1 and PD-L1 blocking antibodies (5 μg/mL) for 12 days. After 12 days, cells were washed and rechallenged with EGCG (25 μM) or cell culture medium and a proliferation readout taken as previously described. (b) Visualization of priming data demonstrating increased number of proliferative wells on inclusion of PD-1 and PD-L1 blockade.

EGCG-specific T-cell clones (TCCs), generated by serial dilution,^10^ were identified in 2 healthy donors positive for HLA-B*35:01 expression (Figure 3A). Drug-reactive T-cells that proliferated in a dose-dependent manner with EGCG were restricted to CD4^+ve^ monoclonal populations (Figure 3B). Clones were observed to secrete both Th_1_ (IFN-γ) and Th_2_ (IL-5 and IL-13) cytokines. Most notably, secretion of Granzyme B was detected in all TCC profiled, indicating involvement of cytotoxic T-cell responses and potential for inducing liver injury (Figure 3C).

**Figure 3:**
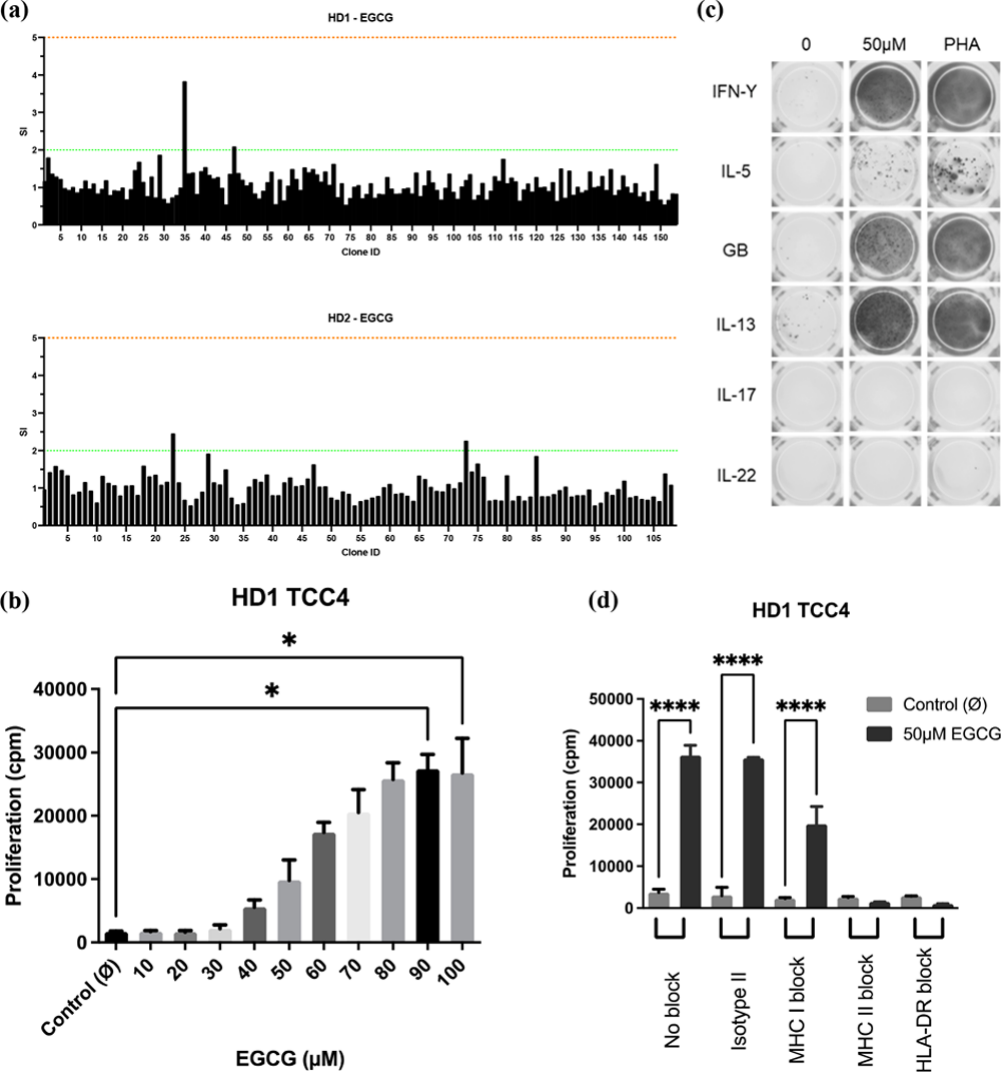
(a) Proliferation of TCC generated from HLA-B*35:01 positive donors (*n* = 2) following treatment with EGCG. TCCs were rechallenged in the presence of APCs with EGCG or cell culture medium for 48 h. Proliferation was measured as previously described. TCCs with a stimulation index (SI) > 1.5 were deemed to be responsive. (b) Dose-dependent proliferation of CD4^+ve^ EGCG-specific TCCs. Clones were incubated with various concentrations of EGCG in the presence of APCs, and proliferation was measured as previously described. Statistical significance was determined using a Kruskal–Wallis test (*p < 0.05). (c) Cytokine secretion of CD4^+ve^ EGCG-specific TCCs. Clones were incubated with EGCG (50 μM) or cell culture medium for 48 h. Secretion of IFN-γ, IL-5, Granzyme B, IL-13, IL-17, IL-22, and Perforin was conducted using an enzyme-linked immunospot (ELISpot) as per the manufacturer’s instructions (Mabtech, Sweden). (d) HLA restriction of EGCG-responsive TCCs. Proliferation in response to EGCG (50 μM) following blockade of HLA complexes by anti-HLA antibodies (10 μg/mL). A proliferation readout was taken as described previously, and statistical significance was determined using a 2-way ANOVA (****p < 0.0001).

TCCs and autologous antigen presenting cells (APCs) were treated with EGCG in the presence or absence of anti-HLA blocking antibodies. Proliferation of TCC was largely unaffected after HLA class I blockade (HLA-A, HLA-B, and HLA-C). However, proliferation was found to be inhibited in the presence of MHC class II blocking antibodies, indicating that T-cell responses to EGCG are driven primarily by MHC class II complexes (Figure 3D). These data suggest that EGCG is able to activate CD4^+ve^ TCC but do not rule out interactions with CD8^+ve^ TCCs, which may be responsible for the small reduction in proliferation seen under MHC Class I blockade, suggesting that MHC Class I may have some role in antigen presentation.

In conclusion, EGCG is demonstrated to be inherently immunogenic, capable of priming PBMCs and T-cells from healthy donors that are positive and negative for the HLA-B*35:01 risk allele. Priming of immune cells demonstrates a dose-dependent trend and is enhanced through the addition of PD-1 and PD-L1 immune checkpoint blockade as demonstrated by *in vivo* research by Cho et al. in 2021. EGCG-responsive T-cells displaying a CD4^+ve^ phenotype were generated from 2-drug naïve healthy donors expressing the HLA-B*35:01 risk allele, associated with GTE-DILI. We have been able to identify TCCs that proliferate and secrete both cytotoxic and inflammatory cytokines such as IFN-γ and Granzyme B, suggesting these CD4^+ve^ T-cells may be responsible for the tissue damage seen. T-cell responses observed in this study were largely driven through MHC Class II (HLA-DR) interactions and not reliant upon expression of the MHC Class I allele association to respond. These data however do not exclude the possibility that CD8^+ve^ TCCs may be present in small numbers or in the tissue resident compartment in patients and contribute to the clinical phenotype seen.

Further investigations into the allele restriction of EGCG responses are warranted, with cloning experiments focusing on individuals negative for HLA-B*35:01 expression needed to identify whether the generation of TCCs is possible in donors not expressing the risk allele. Additional functional T-cell analysis will be required to determine the mechanism by which EGCG is presented to T-cells in a processing-dependent or -independent manner. Further studies are ongoing to identify CD8^+ve^ TCCs from healthy donors, and work is ongoing to recruit GTE-DILI patients to the study.

## Abbreviations

APCs: antigen presenting cells
DILI: drug-induced liver injury
HDS: herbal and dietary supplement
HLA: human leukocyte antigen
PBMCs: peripheral blood mononuclear cells
SI: stimulation index
TCC: T-cell clone
GTE: green tea extract
EGCG: epigallocatechin-3-*O*-gallate
MHC: major histocompatibility complex
PD-1: programmed cell death protein 1
PD-L1: programmed cell death ligand 1
